# Unemployment in Rural Europe: A Machine Learning Perspective

**DOI:** 10.1007/s12061-022-09464-0

**Published:** 2022-06-02

**Authors:** Mehmet Güney Celbiş

**Affiliations:** 1 Department of Economics, Yeditepe University, Istanbul, Turkey; 2 Maastricht Economic and Social Research Institute on Innovation and Technology (UNU-MERIT), Maastricht, The Netherlands

**Keywords:** Machine learning, Rurality, Unemployment, Youth, SHAP values

## Abstract

This paper aims to provide policy-relevant findings that can contribute to the resilience of rural regions by discovering the main individual-level factors related to unemployment in those areas through the use of a set of machine learning techniques. Unemployment status is predicted using tree-based classification models: namely, classification tree, bootstrap aggregation, random forest, gradient boosting, and stochastic gradient boosting. The results are further analyzed using inferential techniques such as SHAP value analysis. Results suggest that access to training programmes can mitigate the labor market inequalities caused by differences in education levels, gender, age, alongside with parental education levels. The results also show how such inequalities are even larger for various subgroups detected by the employed algorithms.

## Introduction

Rural labor markets are perpetually in transformation, resulting from the incessant and rapid evolution of their industrial compositions (Lindsay et al., 2003). Consequently, the ways of approaching the issue of rural unemployment, and the remedies proposed, have been context-sensitive. There were times when unemployment in rural areas was attributed to drastic changes in seasonality patterns of agricultural activities arising from advances in technology and the availability of capital (Gash, 1935). More recent views on the other hand, have drawn attention to network effects, individual employability assets (e.g. basic and key skills, personal characteristics), use of ICT, business size, and accessibility constraints posed by the remoteness from urban markets (Lindsay et al., 2003; McQuaid & Lindsay, 2003; McQuaid et al., 2004; Halden et al., 2005; Jones, 2004). Further interest on disadvantages of rural populations was rekindled in the recent years due to political outcomes related to rural populism which is rooted in the urban-rural disparities and the marginalization of rural populations (Wuthnow, 2018; Bernard et al., 2019). However, not all rural areas are equally subject to the negative effects of the urban-rural divide. For instance, while advanced urbanization benefited rural labor markets that are adjacent to cities, remote rural populations were left behind (Bock, 2018; Bernard et al., 2019). The urban-rural divide has become even more relevant, particularly due to the pandemic conditions. For example, a recent study by Türk (2021) shows that the digital divide between the rural and urban areas has created unequal access to distance education opportunities during the COVID-19 pandemic.

Apart from leading to direct negative social effects, rural unemployment also triggers problems indirectly by pushing individuals from rural to urban areas (Zenou, 2011; Lyu et al., 2019). This phenomenon is particularly pronounced in the case of young individuals that face unemployment in rural locations (Cartmel and Furlong, 2000; Jones, 2004; van Twuijver et al., 2020). The attractiveness of urban areas are also a factor for the outmigration of women from rural areas, as the agglomeration of industry in large urban areas often creates favorable conditions for female employment (Özgüzel, 2020; Alonso-Villar & Del Río, 2008). Especially in the global North, such population movements are frequently related to the insufficiency of public and private services and may lead to depopulation and permanent unemployment (Kühn, 2014; Bock et al., 2016; Bock, 2018; van Twuijver et al., 2020). The downward spiral created by the loss of the young labor force often transforms rural locations into concentrations of older people, leading to further challenges in attracting specialist workers (Steiner et al., 2021) Emigration from rural areas, while draining out rural economies, may in turn lead to problems related to congestion in urban areas. On the other hand, while bringing about a multitude of social consequences, unemployment itself is subject to a wide array of socioeconomic factors that range from the macroeconomic scale to the personal level. Against the background of such a broad spectrum of influences, machine learning (ML) algorithms have emerged as techniques that can provide a deeper understanding of sophisticated relationships (Athey, 2018; Harding & Hersh, 2018; Mullainathan & Spiess, 2017; Varian, 2014).

Applications of ML models even on the general topic of unemployment are rare. Some examples are the studies by Xu et al. (2013), Cook and Hall (2017), and Kreiner and Duca (2019) who use techniques such as neural networks and support vector machines, among other methods, for predicting unemployment in the USA. A similar approach is taken by Katris (2019) who used ML methods to predict unemployment rates in a set of European countries, Gogas et al. (2021) who forecast the Euro area unemployment, and the study by Chakraborty et al. (2020) where a set of ML approaches is employed to predict the unemployment rates in seven countries. In an individual-level study, Montanez and Hurst (2020) used smart meter data to predict personal employment status for a set of individuals in Ireland. The present study on the other hand, uses five different ML applications for investigating the mechanisms behind rural unemployment. To be more precise, rural unemployment is predicted through the use of stochastic and non-stochastic gradient boosting machines, random forest and bootstrap aggregated algorithms in addition to a simple classification tree that serves as a root model for them. The findings of these models are further analyzed using an adaptation of Shapley values from cooperative game theory, alongside with partial dependence and clustering techniques. The single tree, bagging, and random forest models can be considered as providing relatively more descriptive results in relation to the research question, while the interpretable techniques that we subsequently employ constitute the empirical side of this paper. The aforementioned algorithms allow us to profile the individuals in rural areas who are unemployed, through the algorithmic assessment and selection from a large set of predictors. Furthermore, this study introduces alternatives to traditional approaches (e.g. econometric methods) frequently used in regional economic research, and contributes to the presently scarce ML applications in the field. Through the use of these techniques, the study aims to explore the individual-level reasons behind rural unemployment, assess the size and direction of the effects related to unemployment, and identify specific subgroups of individuals that are particularly vulnerable to job loss. The study also aims to algorithmically search and discover additional patterns in the data that may be of interest in addressing challenges relating to unemployment in rural locations.

The rest of this study is structured as follows. “The Data” provides a description of the data. “Binary Recursive Partitioning” constructs a classification (decision) tree and discusses its findings. “Bagging Prediction and Random Forest” expands the analytic work to ensemble models and explains the results of a bagging prediction together with the findings of a random forest implementation. “Non-stochastic and Stochastic Gradient Boosting Machine Applications” elaborates on the results of additive sequential tree models; that is to say, the gradient boosting algorithms (both stochastic and non-stochastic). “Interpretable Machine Learning Applications on the Gradient Boosting Results” implements a series of interpretable ML techniques on the results for the purpose of providing more detailed observation-specific results. “Concluding Remarks” discusses the conclusions of the study.

## The Data

Measuring overall unemployment in rural locations – in comparison to urban areas – is relatively problematic, as major rural occupations are often not entitled to unemployment compensation claims which are often used to estimate official rates (Lasley & Korsching, 1984). The representativeness of rural data may be further hampered by other uncertainties that pertain to the measurement official unemployment rates, such as the discouraged worker effect. Furthermore, the commonly used designation “actively searching for a job” used when classifying the unemployed may result in loss information, as employment opportunities are often shared through informal networks (Lasley & Korsching, 1984). As a result, understanding the circumstances that affect rural unemployment in particular necessitates data at the individual level.

The European Social Survey (ESS) series directed by the European Research Infrastructure Consortium (ERIC) collects micro-level data on individual employment status among other themes. This study uses the eighth round of the ESS survey program (ESS8, 2016). In ESS8, interviewees were asked about their employment status. Two of the options presented were related to being unemployed where the respondent could specify whether they are “unemployed and actively looking for job” or “unemployed, but not actively looking for job.” In order to account for the discouraged workers, we merged these two options such that all individuals who have given any one of these two answers are categorized as unemployed. As a result, the dependent variable is constructed through a binary classification if individuals into the “Unemployed” and “Employed” categories. The data set has been subsequently limited to include only the individuals aged 15 to 64 (i.e. the working age population based on the OECD (2020) definition). Retired individuals are excluded from the dataset.

As a means to reduce heterogeneity in the sample that may exist due to significant dissimilarities of institutional structures unobserved in the data, the sample has been restricted to the EU countries that are included in ESS8, Norway, and the UK. Subsequently, the sample is further subsetted so that only the respondents located in rural areas are included.

The full ESS8 data set includes a multitude of variables (i.e. features) with comparatively very sparse observations, leading to costly limitations on the number of individuals that can be included in the analysis. A great number of survey questions, which are unfeasible to list within the text of this paper, can be grouped under general categories such as: political views, opinions regarding social freedoms, views on religion, experiences regarding discrimination, concerns regarding the environment and climate, alongside of the features selected by the models in the present study. Features with missing values greater than 10% have been excluded from the analysis, as collectively they rendered the dataset unusable, causing the prediction models to drop almost all rows. The ESS8 data also comprises many administrative features (e.g. interview duration, edition, production date, etc.), in addition to rows corresponding to refusals to respond. Such observations were also removed. Lastly, for each categorical predictor, all classifications were encoded as binary features.

The above outlined subsetting stages have reduced the full ESS8 data to a sample comprised of 4,622 observations and 958 features. However, as one would expect, only a small portion of these observations (about 8%) were in the “Unemployed” category. This large imbalance can cause the ML algorithms to be biased towards predicting many individuals as “Employed.” This issue has been counteracted by including – instead of the total available number – a random sample consisting of 10% of the employed individuals in the data set, resulting in both a reduction in imbalance and an increase in computational efficiency.^1^ The resulting sample consists of 762 observations and 958 predictors.

Despite the reduction in the full data, the ML models applied in this study analyse a large number of variables. We therefore define only the features that are chosen by the ML algorithms. The full ESS8 data set, accompanied by the code book for all variables is downloadable in the website of the ESS. For comparability, the sample used in our analysis is available for download.^2^ In the following sections, a set of ML models are employed in order to extract information from the aforementioned dataset. In addition to models oriented towards prediction, we also apply model-agnostic interpretable ML techniques to further elaborate on the result, explore hidded effects, and identify disadvantages faced by subgroups of individuals that may be in need of specific policy approaches.

## Binary Recursive Partitioning

With the aim of setting a point of departure for the ML models applied in the present study, we begin by fitting a classification tree through the application of a binary recursive partitioning algorithm on the training data, based upon the Classification and Regression Trees (CART) method by Breiman et al. (1984).^3^^,^^4^ In the classification tree, the criterion of impurity for a node *j* is given by a Gini score $G_{j}={\sum }^{K}_{k=1}p_{jk}(1-p_{jk})$ and $p_{jk} = \frac {1}{N_{j}}{\sum }_{i \in D_{j}}$**1**(*y*_*i*_ = *k*) where *y*_*i*_ is the observed outcome for the *i*’th individual in the training data (*i* = 1,...,*N*), *k* is the class index, and *D*_*j*_ is the set of all individuals that are in the *j*’th node, and *N*_*j*_ is the number of observations in *j* (James et al., 2013; Friedman, 2001).^5^ At each split, a splitting variable *z*_*m*_ from the feature space *Z* = *z*_1_,...,*z*_*M*_ and *m* = (1,...,*M*) alongside with its split value *c* are chosen such that the sum of weighted Gini scores of the nodes produced by the binary partitioning are minimized:

1 $$\min_{m,c} \left[ \frac{N_{j_{1}}(m,c)}{N}G_{j_{1}}(m,c)+\frac{N_{j_{2}}(m,c)}{N}G_{j_{2}}(m,c) \right]$$

where *j*_1_ and *j*_2_ are the two daughter nodes of *j*, and *N* is the number of observations (Friedman, 2001; James et al., 2013; Breiman et al., 1984). The partitioning described in Eq. 1 is not performed if the split does not yield data regions with a total Gini score that is lower that the Gini value for the observations in *D*_*j*_ (James et al., 2013; Friedman, 2001; Breiman et al., 1984).^6^

A full tree produced in this manner may overfit the data and lead to poor out-of sample predictions (James et al., 2013). To cope with this problem, the complexity of a classification tree is penalized through the application of a *K*-fold cross-validation process.^7^ Based on the descriptions by Friedman (2001) and James et al. (2013) and Sutton (2005) this process can be summarized along the following lines: also referred to as “cost complexity pruning” or “weakest link pruning” the complexity reduction process is initiated by the generation of an unrestricted classification tree *R* and a series of nested subtrees $r \subseteq R$ where the complexity level of each *r* is represented by the parameter *γ* ≥ 0. Greater values of *γ* indicate lower tree complexity such that in fact each subtree of *R* is in turn also nested in the immediate more complex subtree. Following the random partitioning of the training data into *Q* equally sized folds (*q* = 1,...,*Q*), the prediction is performed *Q* times by using each *q* internally as the validation set in each *q*’th round. The parameter of complexity *γ* corresponding to the lowest weighted aggregate misclassification error contingent on *γ*|*r*| (the penalizing term) is determined for each *r* where |*r*| is the number of the leaves of the classification tree (i.e. the number of terminal tree nodes $\bar {j}$):

2 $$\begin{array}{@{}rcl@{}} E_{q}(\gamma)&=&{\sum}^{|r|}_{\bar{j}} e_{\bar{j}}\frac{N_{\bar{j}}}{N}+\gamma |r| \\ \text{where} \ \ \ e_{\bar{j}}&=& \frac{1}{N_{\bar{j}}} {\sum}_{i \in D_{\bar{j}}} \textbf{1}(y_{i} \neq k_{\bar{j}}^{*}) \end{array}$$

where *E*_*q*_(*γ*) expresses the error of subtree *r* in predicting the observations in fold *q* using all other *Q* − 1 folds adjusted by the complexity of *r*, $k_{\bar {j}}^{*}$ is the class that is in majority in the terminal node $\bar {j}$, **1** denotes the indicator function, and $\bar {j}=\{1,...,|r|\}$. Next, the complexity-adjusted subtree errors are averaged for each level of *γ* over the *Q* predictions performed on the left-out folds. The *γ* value indexing the *r*’th subtree with the lowest complexity-adjusted error is:

3 $$\begin{array}{@{}rcl@{}} \gamma^{*}=\underset{\gamma}{{\arg\min}} \left[ \frac{1}{Q} {\sum}_{q=1}^{L} E_{q}(\gamma) \right] \end{array}$$

Finally, from among the initially generated subtrees using the full training data, the subtree $r^{*} \subseteq R$ with a corresponding complexity value *γ* = *γ*^∗^ is used as the final tree model. (Friedman, 2001; Sutton, 2005; James et al., 2013).^8^ In addition, the maximum depth of a node, and the minimum size of a node are determined through a grid search process.^9^ As a consequence, a tree with a maximum node depth and a minimum node size of 5, and a *γ* parameter of 0.02 is grown. The test data accuracy of the resulting classification tree is 61*%*.

**Fig. 1:**
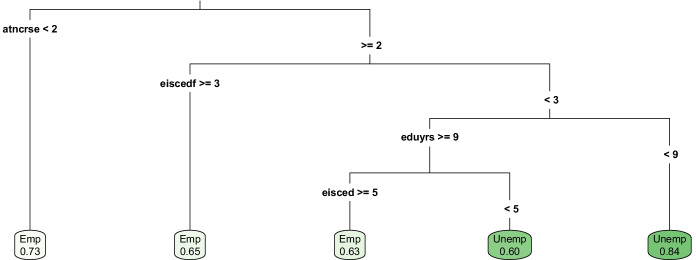
Individual Classification Tree

The predictors selected by the binary recursive partitioning algorithm are defined in Table 1, and the classification tree is shown in Fig. 1. For each terminal node, the share of the predicted category is displayed. The single tree is dominated by predictors related to education. Individuals in rural areas who have taken steps to improve their skills and knowledge – such as by attending courses and training programs (represented by the variable atncrse) – are predicted to be employed. Alongside attendance to such activities, results imply that the skills and knowledge needed for employment may be transferred through personal relationships; an individual is predicted to be employed if the level of education of her/his father (eiscedf) is at least “upper secondary.” This is not an implausible finding; Lindsay et al. (2003) note in their interview-based study that the work record of an individual’s father – which may also be linked to his level of education – can be a very important determinant of employment status in remote rural labor markets. For the group of individuals whose fathers are less educated (i.e. eiscedf< 3) only those with minimum nine years of education who hold at least an advanced vocational degree are predicted as employed.

While the above initial findings related to training and education may seem trivial, they are particularly relevant in the rural context. Chandler (1989) and Cartmel and Furlong (2000) had highlighted the insufficiency of training facilities, specifically for young individuals, in selected rural locations in England and Scotland respectively. Bock (2004) on the other hand, shows how women in particular are affected by the inadequacy of training opportunities in rural Europe. The ML techniques presented in the following sections, particularly the interpretable ML methods, discover and assess the effect directions and sizes of a larger set of features and examine the interactions between them.

**Table 1:** Algorithmically Selected Predictors - Classification Tree (out of 958 Features)

Name	Description	Values
atncrse	Binary variable indicating whether the respondent has taken any course or attended any conference or lecture to improve her/his knowledge or skills for work.	**1:** Yes. **2:** No.
eduyrs	Years of full-time education completed by the respondent.	Numeric predictor.
eisced	A 1 to 7 scale measuring the highest education level attained by the survey participant.	**1** indicates “less than lower secondary” and **7** indicates “Master’s level or more”.
eiscedf	A 1 to 7 scale measuring the highest education level attained by the survey participant’s father.	**1** indicates “less than lower secondary” and **7** indicates “Master’s level or more”.

Some of the above definitions may be identical to those in the ESS8 codebook (ESS8, 2016)

## Bagging Prediction and Random Forest

The sole tree presented in Fig. 1 is merely one possible representation of the multitude of classification trees used either in an ensemble or a sequential manner by the more sophisticated ML algorithms employed in the present study. This process, however, can yield very dissimilar trees when the underlying data set is modified (e.g. by resampling the training and test samples), and leave out important predictors if there is a high degree of correlation among the features (Athey and Imbens, 2019; James et al., 2013). In a bagging prediction, developed by Breiman (1996) and also called the bootstrap aggregation algorithm, multiple classification trees are combined. This is done by drawing, with replacement, a large number of random samples from the training data and constructing a corresponding classification tree *t* for each of those *T* samples (1,...,*T*). The bagging prediction for individual *i* is the majority vote of the ensemble made of *T* classification trees. The optimum number of trees (*T*) is determined by using the estimate for the classification test error, calculated through the prediction of out-of- bag (OOB) observations corresponding to each *t* (James et al., 2013). We have identified the number of trees that yield the lowest classification test error as 16 after making bagging predictions that consist of minimum 10, and maximum 500 classification trees. The accuracy of this bagged model is 63.5*%*.

**Fig. 2:**
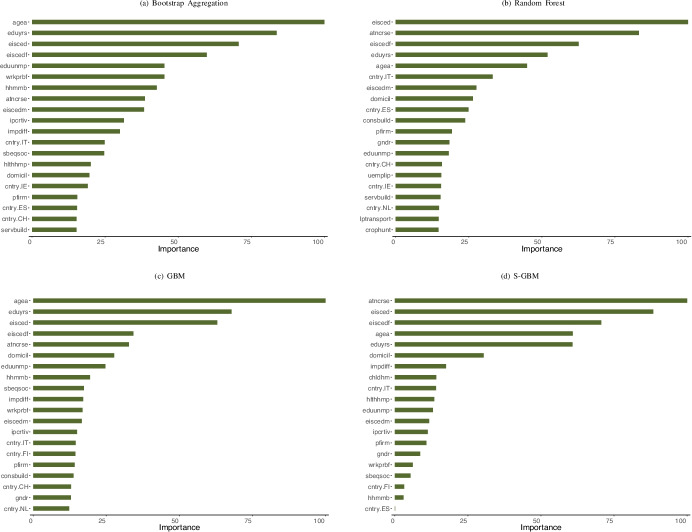
Variable Importance Plots

The bootstrap aggregation method can be also used to calculate the variable importance values. The overall decrease in node impurity resulting from the usage of a given predictor as the splitting feature, presented in Eq. 4, is averaged over the *T* trees and scaled into an interval ranging from 1 to 100 (James et al., 2013):

4 $$\begin{array}{@{}rcl@{}} {\Delta} G = G_{j}-\left[ \frac{N_{j1}}{N}G_{j1}+\frac{N_{j2}}{N}G_{j2} \right] \end{array}$$

where *G*_*j*_ is the Gini value of the parent node. The most important 20 predictors identified by the bootstrap aggregation algorithm and their definitions are presented in Fig. 2a and Table 2 respectively (the variables already defined in “Binary Recursive Partitioning” are not included). The findings of the bagged prediction is discussed in combination with the results of the below outlined random forest application.

The random forest algorithm developed by Breiman (2001) instills extra randomness into each tree – in addition to the random sampling employed by bagging – for the purpose of reducing the correlation between the trees in the ensemble (James et al., 2013; Friedman, 2001). When the splitting of a node is attempted in a random forest tree, the set of variables considered is restricted to a random subset *L* ⊂ *Z*, and |*L*| is generally taken as $\sqrt {|Z|}$ in the classification context (Friedman, 2001; Breiman, 2001). Our random forest algorithm (with 500 trees) performs with an accuracy level of 65*%*. The variable importance degrees are presented next to those of the bagging prediction in Fig. 2b. While random forest and bagged predictions confirm the importance of training and education related factors, both models highlight the role of age in predicting rural unemployment. For the bootstrap aggregation algorithm, the age of an individual has been the predictor that provides the highest reduction in node impurity. The random forest application on the other hand, also reports age as one of the top predictors. In general, the two ensemble learning methods provide further information on several other features that were not selected by the classification tree. For instance, a person’s opinions on the importance of being creative, the number of people in the household, and being residents of Italy or Spain are also important predictors. Regarding the findings on Italy and Spain, partial dependence plots suggest that individuals in the rural areas of these countries are more likely to be unemployed.^10^

**Fig. 3:**
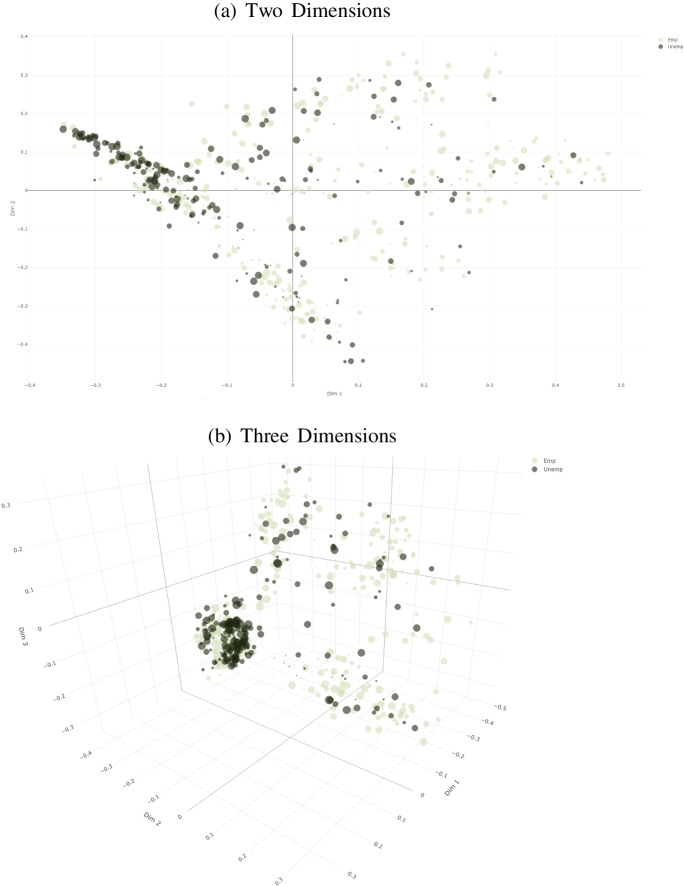
Proximity Plots: Random Forest

Further information can be harnessed from the random forest results by generating a matrix of proximities between observations and plotting these proximities in reduced dimensional forms (Friedman, 2001; Breiman and Cutler, 2020). The employment status of the individuals that are excluded from a given classification tree *t* within the random forest (i.e. those that are the OOB observations for tree *t* as outlined earlier) are predicted by *t*, and each time in an iteration *t* the predicted outcomes of two OOB observations *i* and *l* (*l* = 1,...*N*, *i*≠*l*)^11^ fall into the same terminal region $D_{\bar {l},t}$, the element *a*_*il*_ of the initially zero *N* × *N* matrix **A** is increased by 1 (Friedman, 2001; Breiman & Cutler, 2020; Aldrich & Auret, 2013). **A** is subsequently divided by the number of trees in the random forest |*T*| and expressed in terms of dissimilarities in the form of a matrix **P**= 1 −**A** (Aldrich & Auret, 2013). The visualization of the information contained in **P** in lower dimensions (typically three or two) is done through metric multidimensional scaling (Friedman, 2001). The resulting “random forest proximity plots” are presented in Fig. 3a and b in two and three dimensions respectively. In the proximity plots, the darker circles indicate the unemployed individuals vis-à-vis the lighter colored which indicate the employed persons. The larger a circle, the older is the corresponding individual. Visualizing proximities between observations as outlined above also allows for the detection of clusters (Friedman, 2001; Aldrich & Auret, 2013). Both proximity plots suggest that the random forest algorithm has been able to separate the two categories to a certain extent; clusters of unemployed individuals are hinted on the left-hand-sides of both plots.

**Table 2:** Algorithmically Selected Predictors - Random Forest and Bagging (out of 958 Features, Cont’d)

Name	Description	Values
agea	Age of the participant.	Numeric predictor.
chldhm	Categorical feature indicating whether there are children living in the respondent’s household.	**1:** Yes. **0:** No.
cntry.	The participant’s country of residence (followed by the country abbreviation). Binary variable.	Equals 1 if the participant resides in the country and 0 otherwise.
consbuild	Binary variable indicating whether the respondent’s current or past main job or the kind of work she/he did or does most of the time is defined as “construction of buildings.”	**1:** Yes. **0:** No.
crophunt	Binary variable indicating whether the sector of the respondent’s current or past main job is defined as “crop and animal production hunting and related service activities.”	**1:** Yes. **0:** No.
domicil	Binary variable categorizing the type of the rural area where the respondent lives in.	Types are: “Country village,” “Home or farm in countryside.”
eduunmp	Score indicating the how strongly the respondent is in favor of the government spending more on training and education programs for unemployed individuals at the cost of a reduction in unemployment benefits.	A scale of 1 to 4 where **1** is “strongly against” and **4** is “strongly in favor.”
eiscedm	A 1 to 7 scale measuring the highest education level attained by the survey participant’s mother.	**1** indicates “less than lower secondary” and **7** indicates “Master’s level or more”.
gndr	Gender of the participant.	Binary predictor.
hhmmb	Number of regular members of the household.	Numeric predictor.
hlthhmp	Score measuring how badly by the respondent’s daily activities are hampered by health and/or disability related problems.	A scale of 1 to 3 where **1**: “Very much” and **3**: “Not at all.”
impdiff	Willingness of the respondent to try different and new things in life.	A scale of 1 to 6 where **1** means strong willingness and **6** means strong unwillingness.
ipcrtiv	Score measuring the level of importance that the respondent places on being creative and seeking new ideas.	A scale of 1 to 6 where **1** signifies very high importance and **6** signifies very low importance.
lptransport	Binary variable indicating whether the sector of the respondent’s current or past main job is defined as “land transport and transport via pipelines.”	**1:** Yes. **0:** No.
pfirm	Binary variable indicating whether the respondent has worked or is working in the private sector.	**1:** Yes. **0:** No.
sbeqsoc	Score measuring the extent of agreement by the respondent that the social services and benefits in the country lead to a higher degree of equality in the society.	A scale of 1 to 5 where **1**: “Agree strongly” and **5** “Disagree strongly.”
servbuild	Binary variable indicating whether the respondent’s current or past main job or the kind of work she/he did or does most of the time is defined as services to buildings and landscape activities.	**1:** Yes. **0:** No.
uemplip	Binary variable indicating whether the respondent’s partner has been unemployed and not looking for a job during the last seven days.	**1:** Yes. **2:** No.
wrkprbf	Score indicating the how strongly the respondent is in favor of the government introducing extra services and social benefits directed towards working parents to combine family and work life at the cost of much higher taxes for all.	A scale of 1 to 4 where **1** is “strongly against” and **4** is “strongly in favor.”

Some of the above definitions may be identical to those in the ESS8 codebook (ESS8, 2016)

## Non-stochastic and Stochastic Gradient Boosting Machine Applications

The ensemble methods presented in the previous section uncovered useful information on rural unemployment using information from large collections of separately grown trees. Sequential models on the other hand, allow the classification trees to learn from past prediction errors. Two sequential models that we apply in this study are the gradient boosting machine (GBM) developed by Friedman et al. (2001), and its extension, the stochastic gradient boosting machine (GBMS), established by Friedman (2002). The gradient boosting algorithms differ from the random forest and bootstrap aggregation models by growing the trees sequentially and attributing greater weights on individuals whose employment status were previously misclassified. Following Friedman (2001) and Friedman (2002) and Friedman et al. (2001), we construct a sequence of gradient boosted trees and compute the final predictions by implementing the following procedure: Firstly, the negative likelihood of the loss function *L*(*y*_*i*_,*f*(*Z*)) is minimized with respect to the logarithm of odds of being unemployed *f*(*Z*), and the resulting value is used as the approximation of the initial $\hat {y}_{i,t}$ for all *i* individuals collectively:

5 $$\hat{y}_{i,1}=\underset{f(Z)}{\arg\min} {\sum}_{i=1}^{N} L(y_{i},f(Z))$$

To allow a gradient boosted tree *t* belonging to sequence 1,...,*T* to partially correct the errors made by its preceding counterpart *t* − 1, the errors of the latter is expressed in the form of pseudo-residuals *𝜖*_*it*_ for each person *i* in the training data:

6 $$\epsilon_{it} = - \left[ \frac{\partial L(y_{i},f(Z_{i})) }{\partial f(Z_{i})} \right]_{f=f_{t-1}} for \ \ i=1,...,N$$

Subsequently, a regression tree is fitted to predict the pseudo-residuals.^12^ The tree generates terminal nodes $\bar {j}$ comprised of the residuals of all *i* individuals $Z_{i} \in D_{\bar {j}}$. The output of a terminal node $\epsilon _{\bar {j}r}$ is given by Friedman (2001) and Friedman et al. (2001):

7 $$\epsilon_{\bar{j}r} = \underset{\epsilon}{\arg\min} {\sum}_{z_{i} \in D_{\bar{j}r}} L(y_{i},f_{t-1}(Z_{i})+\epsilon)$$

Using the predicted errors of the previous tree, the subsequent tree then learns, to some degree, from the predictions *f*_*t*− 1_ made in the previous iteration, and revises the predictions for individual *i* accordingly:

8 $$\hat{y}_{it}=f_{t-1}(Z_{i})+\alpha \epsilon_{\bar{j}r}\boldsymbol{1}(Z_{i} \in A_{\bar{j}r})$$

where the degree of learning, also referred to as the learning rate, is denoted by *α*, such that $\alpha \epsilon _{\bar {j}r}$ is the improvement made by tree *t* on the predictions of tree *t* − 1, and **1** is the characteristic function (Friedman et al., 2001; Friedman, 2001). The adjustment done by tree *t* is itself limited (0 < *α* < 1) as improvements of a given tree may also be subject to errors. In fact, a low *α* combined with a large number of sequential trees is shown to improve predictions (Friedman, 2001; Friedman et al., 2001). We implement the GBMS model using the programming enhancements provided in the Extreme Gradient Boosting Machine algorithm (Xgboost) developed by Chen et al. (2015) which enables the user to introduce further regularization (such as pruning) of the classification trees as outlined in “Binary Recursive Partitioning”. Xgboost also makes it possible for the GBM to incorporate additional stochasticity through the selection of a random sample when constructing each tree in addition to selecting a random subset of variables *X* ⊂ *Z* at each binary split in each tree in the sequence, similar to the random forest approach.

In order to cope with potential overfitting caused by too complex trees, Friedman (2002) suggests the usage of a learning rate (i.e. shrinkage parameter) of 0.005, a random sample proportion of 50*%*, and maximum number of leaves (terminal nodes) of 6. In addition, we limit the predictors considered at each split to a subsample corresponding to 50*%* of the full set of features, set the minimum $N_{\bar {j}}$ to 5, and the maximum iteration number to 5000. The resulting GBM and GBMS algorithms are 66.2*%* and 61.4*%* accurate in predicting the employment status of the individuals in the test data. Figure 2c and d present the variable importance levels assessed by the two models. The sequential learning results are fairly consistent with those of the earlier applied ensemble tree algorithms. The variables pertaining to education related variables and age are selected as top predictors by both the GBMS and the GBM. However, the variable importance values presented in the previous sections provide information only on the importance of a feature for achieving the best possible predictions, and they do not provide information on how age, education, or any of the remaining features are related to employment status. In this regard, we elaborate on the relationship between a feature and the predicted employment status using partial dependences (Friedman, 2001) and Shapley value methods in the next section.

## Interpretable Machine Learning Applications on the Gradient Boosting Results

Interpretable ML methods allow us to retrospectively understand how a variable is related to the outcome after the predictions are generated. We examine our results using partial dependences, conditional expectations, and Shapley values. The first of these approaches, the partial dependence plots, are constructed for typically two variables at most by grouping the features of interest *Z*_*C*_ and the remaining variables *Z*_*S*_ separately in the training data and computing:

9 $$f_{C}(Z_{C})=\frac{1}{N}{\sum}_{i=1}^{N} f (Z_{C}, Z_{i,S})$$

where the *Z*_*i*,*S*_ are values of variables in *Z*_*S*_ that remain constant, and predictions *f*_*C*_(*Z*_*C*_) are redone for each feature value in *Z*_*C*_ and averaged over individuals *i* (Friedman, 2001; Aldrich and Auret, 2013). While useful as a basic look at relationships between input variables and the outcome, this approach can result in implausible implications for features strongly correlated with variables in *Z*_*S*_ (Friedman, 2001; Molnar, 2019). An additional shortcoming of the partial dependence approach is that the partial effects are averaged, and therefore, individual patterns cannot be observed (Molnar, 2019). As formulated by Goldstein et al. (2015), the disaggregation of the global partial dependence for a feature and plotting the conditional expectations for each observation *i* yields Individual Conditional Expectation (ICE) plots where the heterogeneity across the individuals in the training data can be visualized (Goldstein et al., 2015; Molnar, 2019). However, stacked ICE curves for all individuals *i* in the training data will have separate intercepts. The expression can become visually more informative with regards to the heterogeneity of the effect of the variable of interest for different individuals upon centering all the ICE curves *f*_*i*,*C*_ at a particular anchor point *z*^∗^, and therefore, removing the variation in levels caused by the different *Z*_*i*,*S*_ values by plotting the centered individual conditional expectations $f_{i,C}^{centered}$ (Goldstein et al., 2015; Molnar, 2019):

10 $$f_{i,C}^{centered}=f_{i,C}- f (z^{*}, Z_{i,S})$$

**Fig. 4:**
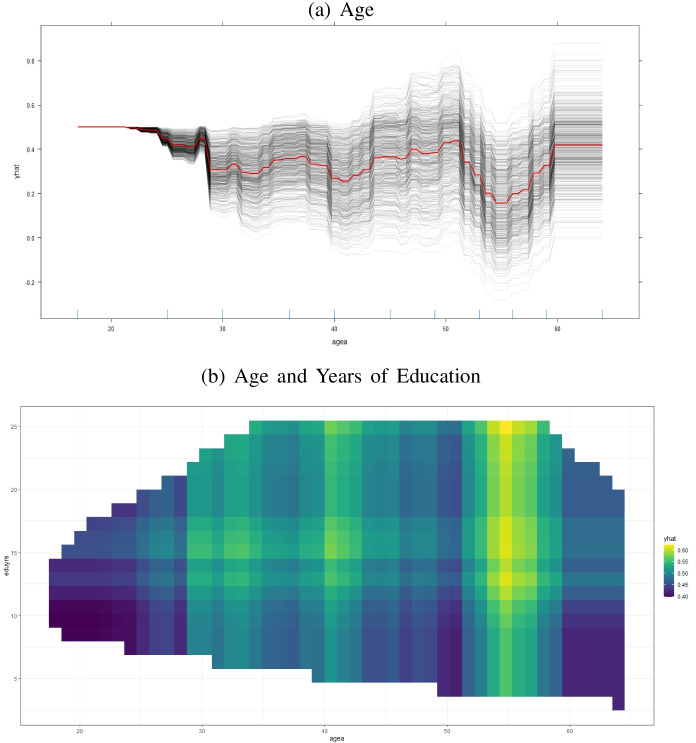
Individual Conditional Expectation Plots: Stochastic Gradient Boosting

Figure 2 displays the centered individual conditional expectation (ICE) graph based on the GBMS model for the feature agea, alongside with the two-way partial dependence plot (PDP) for agea and eduyrs.^13^ Figure 4a indicates that the probability of being unemployed is on average lower for middle-aged persons (shown by the red partial dependence curve), compared to the individuals aged between 16-22 which is within the range defined by the OECD (2020) for youth unemployment (15-24). The probability of unemployment further drops for the individuals between the ages of about 50 and 60. The black ICE curves on the other hand, imply considerable heterogeneity. This observation is an expected outcome; our ML models precisely address this situation among other issues, by allowing for all possible interactions in the data. The bright yellow pixels in the two-way PDP in Fig. 4b represent high probabilities of employment, and are concentrated mostly around the areas that correspond to middle-aged individuals (up to around age 60) and to durations of education that are, approximately, higher then 10 years.

**Fig. 5:**
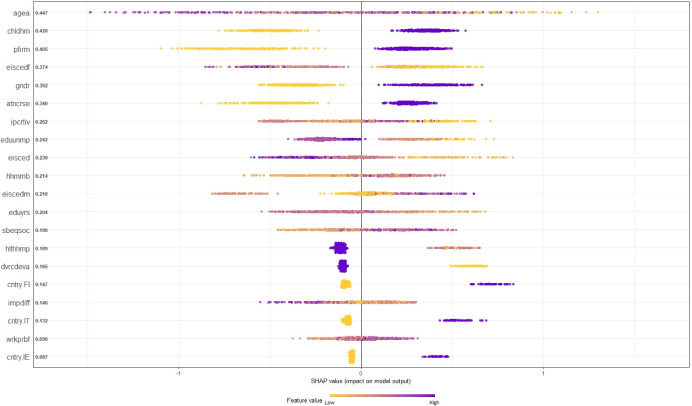
SHAP Values - Extreme Gradient Boosting Machine

The Shapley Additive Explanation (SHAP) method is a novel approach adapted into the machine learning framework by Lundberg and Lee (2017) based on the use of Shapley values from cooperative game theory developed by Shapley (1953). The SHAP approach is able to summarize both the sizes and the directions of the effects of each feature for each data instance. In this regard, the framework presented by Lundberg and Lee (2017) can be summarized as follows: the contribution of the value *v* of a given variable *z*_*m*_ to the deviation of the predicted outcome from the average, for the *i*’th individual, is denoted by *ϕ*_*v*_ and computed as

11 $$\phi_{v}= {\sum}_{S\subseteq Z \backslash\{v\}} \frac{|S|!(|Z|-|S|-1)!}{|Z|!} \left[ g(S \cup \{v\})-g(S) \right]$$

where *S* is a subset of variable values from the features for observation *i* which are to be held constant, while multiple GBM predictions for *i* are calculated by replacing the features except those in *S* by randomly drawn observations from the training data, and subsequently calculating the deviation of the expected value for individual *i* from the average prediction (Lundberg & Lee, 2017; Molnar, 2019).^14^ More specifically, this step is performed by the function *g* as follows:

12 $$g(S)= \int f(Z)dP_{z \notin S}-E(f(Z))$$

whereas in the term *g*(*S* ∪{*v*}) in Eq. 11 the actual feature value for observation *i* is preserved alongside with those in *S*, and the step is repeated for all possible combinations that can produce a subset $S \subseteq Z \backslash \{v\}$ and averaged over all the combinations (Molnar, 2019). As Štrumbelj and Kononenko (2013) and Molnar (2019) point out, calculating multiple predictions with *v* versus with a randomly drawn *v* for all combinations $\frac {|S|!(|Z|-|S|-1)!}{|Z|!}$ is computationally very inefficient. An approximation of the SHAP value for individual *i* is formulated by Štrumbelj and Kononenko (2013) and is presented in Eq. 13:

13 $$\bar{\phi}_{v}= \frac{1}{B}{\sum}_{b=1}^{B} \left[ f^{*}(\hat{y}(i)_{+v}^{b})-f^{*}(\hat{y}(i)_{-v}^{b}) \right]$$

where the outcome for the *i*’th individual ($f^{*}(\hat {y}(i)_{+v}^{b})$) is predicted by replacing a random subset of the variable values for this person while preserving its specific true feature value *v* (hence the subscript + *v*). The prediction is repeated, but this time the value of the variable of interest, *v*, is also replaced by the corresponding value of the randomly drawn observation *l*, and this predicted outcome $f^{*}(\hat {y}(i)_{-v}^{b})$ is subtracted from the prediction that preserves the true *v*. The difference between the predicted outcomes for person *i* with and without *v* is computed in this randomized fashion *B* times (*b* = 1,...,*B*) and the average $\bar {\phi }_{v}$ is calculated (Molnar, 2019; Štrumbelj & Kononenko, 2013). All SHAP outcomes in the present study computed using the R package SHAPforxgboost (Liu & Just, 2020) which also calculates the global SHAP importance levels for every feature and observation by aggregating the absolute SHAP values for a given feature *v* for all *i* and the variables in Fig. 5 are ordered based on their SHAP importance levels computed as (Molnar, 2019):

14 $${\Phi}_{v}={\sum}_{i=1}^{n} |\phi_{i,v}|$$

Figure 5 visualizes the resulting SHAP values of each feature and person. Each individual in the training dataset is represented by a color-filled circle where darker circles indicate a higher *v* (for binary values only two colors are used). The x-axis values indicate the SHAP values. As in all previous importance plots, we report the features with the highest importance scores. In addition to the features selected by the earlier presented models, the SHAP procedure identifies dvrcdeva, a binary feature indicating whether the respondent has ever divorced or had a civil union dissolved, as a relevant variable. Consistent with the earlier findings, the SHAP results suggest that age is a major determinant of rural unemployment. A cluster of light colored circles to the far right in the row for agea (the first row in Fig. 5) indicate that young age has contributed strongly to the prediction of uneemployment for those individuals. The SHAP plot also shows that individuals with children at home (chldhm) – represented by light colored circles – are less likely to be unemployed. However, it is possible that this feature captures the effect of age, as middle-age individuals whom we predict to be mostly employed are more likely to have children at home compared to younger or older persons. The findings also suggest that individuals who work in the private sector (those represented as dark circles in the pfirm row) are more vulnerable to job loss compared to people who are working in the private sector or are self-employed. We also observe that the highest level of education of a person’s father (eiscedf) contributes to the prediction of lower-than-average unemployment probabilities in accordance with our previous results. The SHAP values also underline the disadvantages faced by women in rural labor markets, as gender has contributed positively to the prediction of unemployment for female respondents. One of our main variables of interest that was highlighted by the previous models is atncrse which indicates whether the respondent took steps to improve her/his knowledge and skills by attending a course, lecture, or a conference. The SHAP values show that attending such activities lowered the probability of being unemployed (circles marked with light colors).

**Fig. 6:**
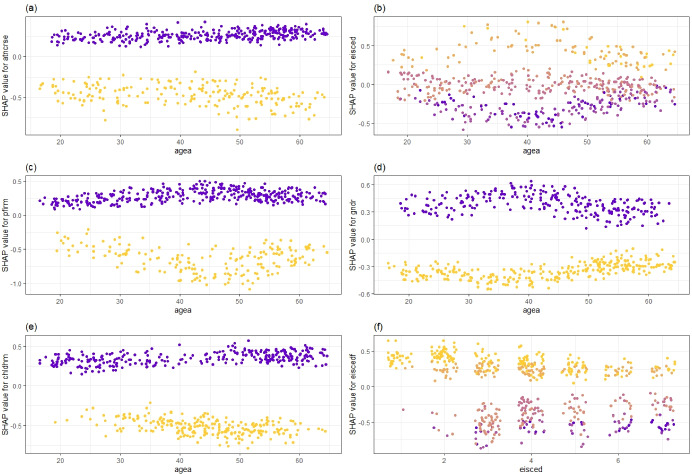
SHAP Interaction Plots - Extreme Gradient Boosting Machine

Instead of assessing the above mentioned major effects in isolation, we examine SHAP interaction plots in Fig. 6 (Molnar, 2019; Liu & Just, 2020).^15^ In panel (a) of Fig. 6 the persons who have not attended a course, lecture, or a conference are represented as dark circles while those who did are marked in light colors. We observe that the SHAP value of this variable is positive if the person did not attend such an activity (adds to the prediction of a person’s status as unemployed) and negative otherwise. A slight divergence between the two groups is suggested to exist as age increases, indicating that such additional/complementary training activities are more important for older individuals with regard to their employment probabilities. In panel (b), we see that high levels of education (dark colors) yield negative SHAP values, where low levels yield positive SHAP values (i.e. increase the prediction of unemployment). Intermediate levels of education, on the other hand, do not seem to have strong effects. The impact of the level of education depends on age, as the discrepancies are larger for middle aged individuals, whereas they are less pronounced for younger and older groups.

In panel (c), persons who work in private firms are represented with dark colors, and those who work in the public sector, or are self-employed are shown in light colors. We observe that people who are employed in private firms are particularly vulnerable to unemployment, and that this vulnerability is a higher concern for middle-aged persons. We also observe a similar result for gender, as shown in panel (d) where dark colored circles represent females and light colors represent males. The plot suggests that being female makes a big difference as the SHAP values of women are all above zero. This difference begins to narrow to some extent for persons older than 50 years old. People without children are attributed higher SHAP values (panel (e)), but as earlier noted, this finding may also be capturing the effect of the variable age. Finally, panel (f) shows how a persons own education interacts with the father’s education level which was observed to be an important feature yielding high SHAP values. Persons with low levels of father’s education (light colors) have positive SHAP values, but this disadvantage in the labor market is mitigated to some extent as the person’s own education level increases.

**Fig. 7:**
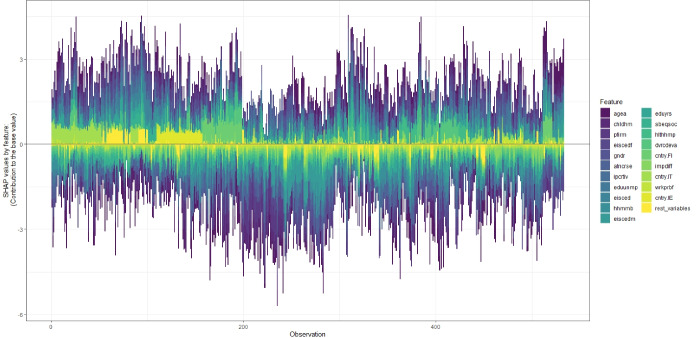
SHAP Force Plot - Extreme Gradient Boosting Machine

Finally, the SHAP force plot (Molnar, 2019; Liu & Just, 2020) presented in Fig. 7 shows that the top 20 variables with the highest SHAP importance levels account for most of the variation in the SHAP value calculations for each individual (observation) represented by a bar. The additive contribution to the individual specific SHAP value of all remaining features for a given person is grouped under the category “rest_variables” and shown in bright yellow. The force plot suggests that reporting the top 20 variables has been a reasonable approach in our models.

## Concluding Remarks

As an issue that is hard to detect, poverty and unemployment in rural areas attracts relatively less political interest in Europe (Chandler, 1989; Bernard et al., 2019). This being said, unemployment in rural Europe has often been identified as a persistent problem particularly for young individuals (Philip & Shucksmith, 2003; Unay-Gailhard, 2016).

This study provided new evidence with regard to the recent state of rural unemployment in Europe by using up-to-date statistical learning methods. In particular, the research question has focused on understanding *who* are the individuals facing difficulties in finding jobs in rural locations, and why they are unemployed while others are not. The results suggest that issues related to lack of adequate training and education opportunities, and youth unemployment in rural areas – highlighted over the decades in many studies – still persist. In addition to features related to age and education, the algorithms have selected other predictors related to a person’s character and household attributes. Further, in depth empirical assessments of the findings showed that persons who come from lower educated families (as shown by the father’s education variable), face difficulties in becoming competitive in the labor market, and even for those who have high levels of education themselves this disadvantage is not reduced sufficiently. This result may be related to personal networks and connections which may be weak for even educated persons in rural areas if their fathers, due to low education, were excluded from such circles. Therefore, policies that aim to increase the inclusiveness of children of disadvantaged families, by increasing their chances to match with available jobs in the rural labor markets may present benefits.

We have also observed that while attending complementary training activities are particularly helpful for reducing a person’s probability to be unemployed, some evidence suggests that elderly people may benefit the most from such activities. Whereas the general level of education affects employment status, the effect is much more noticeable for persons roughly above the age 30 and below 50 years old. This implies that education does not necessarily provide a way out of unemployment for persons younger that 30 years old, and that the benefit of having a higher level of education is limited for younger persons. The employment discrepancies become particularly pronounced in the case of women, who face clear disadvantages. Establishing or enhancing job placement assistance programs for young persons and women in rural areas may potentially generate welfare-increasing outcomes. We also observe that job security is a concern particularly for persons working in private firms as they are more prone to become unemployed compared to other groups (this finding is especially relevant for middle-aged persons). Therefore, legal improvements aimed to increase job security levels in rural markets may provide further ameliorations to the living standards of the rural workforce. Finally, some country-level effects are found (such as for the cases of Italy and Spain) which call for thorough case studies on the rural labor markets in these countries.

## Footnotes

1 Searching for the best tuning parameters to be used in the ML algorithms, through running grid searches using average home computers, can take several days.

2 For replication purposes, the dataset and the code can be downloaded from the folowing links: https://drive.google.com/file/d/1G14AHu12c8ngLERsbyL7P-pBT7y18pxP/view?usp=sharing and https://drive.google.com/file/d/1TDTzHJCNeNtj4hyrcYf7u0LYqwhkT1Df/view?usp=sharing. The complete ESS8 data set and the code book including all feature definitions is available at: https://www.europeansocialsurvey.org/data/multilevel/guide/bulk.html.

3 The training data corresponds to the randomly sampled 70*%* of the complete data, and the test data consists of the remaining thirty percent.

4 The R functions used in this study are: randomForest by Liaw and Wiener (2002) for random forest and the associated proximity matrix, gbm by Ridgeway (2004) for the gradient boosting machine, Xgboost by Chen et al. (2015) for the stochastic gradient boosting machine, train by Kuhn (2008) for the bootstrap aggregated model, rpart by Atkinson and Therneau (2000) for binary recursive partitioning, rpart.plot by Milborrow (2019) for visualizing the classification tree, pdp by Greenwell (2017) for the individual conditional expectation graphs, shapforxgboost by Liu and Just (2020), and ggplot2 by Wickham (2011) for graphs.

5 Since the dependent variable has a two-class structure, *G*_*j*_ is equal to 2*p*_*j*1_(1 − *p*_*j*1_).

6 If *z* is a categorical feature, *c* separates the variable into classes or sets of classes.

7 In order to avoid confusion with the class index which is also denoted by *k*, we index the number of data folds in the cross-validation step by *q*. The term “internal” is used to distinguish a given fold *q* from the test data which is left out earlier.

8 It is possible that the cross-validation may indicate that the optimal tree is the unrestricted *R* (i.e. *γ*|*r*| = 0 in Eq. 2). Therefore, the set of subtrees is expressed as “$r^{*} \subseteq R$” as opposed to “*r*^∗^⊂ *R*” (James et al., 2013).

9 The depth and size parameters were iterated by increments of 1 in a range of two to twenty.

10 The partial dependence plots for these variables are not reported in this study and can be provided if requested.

11 The second observation is indexed by *l* instead of the convenient *j* for avoiding confusion with the tree nodes *j*.

12 As opposed to a classification tree, the binary criteria used in the recursive partitioning steps performed by a regression tree is often a loss function with a squared error form instead of node impurity. Regression trees, not covered in the present study, are formulated by Breiman et al. (1984) within the CART structure alongside with classification trees.

13 Partial dependence plots are developed by Friedman (2001), and the individual conditional expectation plot is developed by Goldstein et al. (2015).

14 The cardinality of *Z* as denoted in Eq. 11 is an alternative way to express the total number of features *M* as defined in “Binary Recursive Partitioning” (|*Z*| = *M*).

15 A low degree of jitter is used in the plots for better readability.
